# Altered Passive Knee Kinematics in Type 2 Diabetes Mellitus Revealed by Pendulum Test and Model-Derived Viscoelastic Parameters

**DOI:** 10.3390/s26185884

**Published:** 2026-09-17

**Authors:** Maria Stella Valle, Gianfranco Bosco, Paolo Andò, Salvatore Auteri, Cristina Russo, Maria Teresa Cambria, Sofia Surdo, Lucia Malaguarnera, Giuseppe D’Arrigo, Matteo Cioni

**Affiliations:** 1 Laboratory of Neuro-Biomechanics, Section of Physiology, Department of Biomedical and Biotechnological Sciences, School of Medicine, University of Catania, 95123 Catania, Italy; maria.valle@unict.it; 2 Department of Systems Medicine and Centre for Space BioMedicine, University of Rome Tor Vergata, 00133 Rome, Italy; gianfranco.bosco@uniroma2.eu; 3 Movimento in Salute Onlus APS, 95014 Giarre, Italy; andopao@gmail.com; 4 Unit of Vascular Surgery, ARNAS Garibaldi-Nesima Hospital, 95122 Catania, Italy; s.auter@tiscali.it (S.A.); gdarr1962@gmail.com (G.D.); 5 Section of Pathology, Department of Biomedical and Biotechnological Sciences, University of Catania, 95123 Catania, Italy; cristina.russo@unict.it (C.R.); lucmal@unict.it (L.M.); 6 Section Medical Biochemistry, Department of Biomedical and Biotechnological Sciences, University of Catania, 95123 Catania, Italy; cambrimt@unict.it; 7 Italian Center for the Study of Osteopathy (CSDOI), 95124 Catania, Italy

**Keywords:** type 2 diabetes mellitus, knee biomechanics, instrumented pendulum test, viscoelastic properties, joint stiffness, joint viscosity

## Abstract

Background: Type 2 diabetes mellitus (T2DM) is associated with musculoskeletal alterations that may affect joint mechanics, yet quantitative characterization of passive knee behavior remains limited. This study investigated passive knee mechanics in individuals with T2DM using an instrumented pendulum test combining kinematics with biomechanical modeling. Methods: Twenty matched participants (10 with T2DM, 10 controls) underwent ten pendulum test trials of the right lower limb under electromyographic monitoring. Primary kinematic outcomes included flexion and extension amplitudes, angular velocities, relaxation indices, and oscillation duration, whereas model-derived stiffness and viscosity were secondary outcomes. Participant-level kinematic outcomes were compared using Wilcoxon rank-sum tests with Benjamini–Hochberg false discovery rate correction. Stiffness and viscosity across successive flexion–extension half-cycles were analyzed using aligned rank transform ANOVA models including Group, flexion-extension Half-Cycle, and their interaction. Results: Compared with controls, participants with T2DM showed reduced normalized first flexion and extension amplitudes and reduced extension velocity, whereas first flexion velocity was not significantly changed. Plateau amplitude and oscillation duration also differed between groups, while relaxation indices did not. Model-derived knee stiffness and viscosity showed no significant overall Group effect but varied across half-cycles and exhibited significant Group × Half Cycle interactions. However, post hoc comparisons did not identify significant between-group differences at corresponding individual Half-Cycles after correction for multiple comparisons. Conclusions: Individuals with T2DM exhibited differences in passive knee behavior, primarily characterized by reduced oscillatory excursions and altered temporal characteristics. Exploratory analyses of model-derived stiffness and viscosity indicated differences in their phase-dependent patterns across the pendular response, although no specific half-cycle showed a significant between-group difference. These exploratory findings require confirmation in larger cohorts. Instrumented pendulum testing may provide complementary, non-invasive information on system-level passive knee kinematics beyond conventional clinical assessment.

## 1. Introduction

Type 2 diabetes mellitus (T2DM) is a chronic metabolic disorder associated with a wide spectrum of systemic complications affecting not only vascular and neural tissues but also the musculoskeletal system. Although diabetic musculoskeletal manifestations have historically received less attention than microvascular and neurological complications, they are increasingly recognized as a clinically relevant component of diabetes, involving muscles, tendons, connective tissues, and joints and contributing to impaired mobility and functional disability [1,2]. Among the mechanisms proposed to contribute to diabetes-related musculoskeletal remodeling, chronic hyperglycemia promotes the accumulation of advanced glycation end-products (AGEs) within long-lived extracellular matrix proteins. AGE-mediated non-enzymatic collagen cross-linking can modify collagen fibril organization, intermolecular sliding, energy dissipation, and mechanical behavior [2,3,4]. Recent biomechanical evidence further indicates that the effects of AGE accumulation depend on cross-link density and properties, supporting a more complex relationship between glycation and tissue mechanics than a uniform increase in stiffness [4].

In musculoskeletal tissues, diabetes-related structural changes have been associated with altered soft-tissue mechanical and viscoelastic properties, reduced tissue compliance, and impaired joint mobility [1,2]. Because passive joint behavior depends on the mechanical contribution of muscles, tendons, ligaments, and connective tissues, articular structures, and segmental inertia, diabetes-related alterations in tissue composition may influence the mechanical response of joints even in the absence of voluntary muscle activation.

The knee joint represents a suitable experimental model for investigating passive mechanical behavior because its motion during pendular testing results from the interaction between gravitational forces and passive soft-tissue constraints. The pendulum test, originally introduced by Wartenberg [5], offers a non-invasive method for assessing passive lower-limb dynamics by analyzing the free oscillation of a fully relaxed limb under gravity. Subsequent biomechanical studies have demonstrated that pendulum oscillations can provide quantitative information on passive joint behavior, including oscillation amplitude, relaxation characteristics, and mechanical parameters derived from damped second-order system models [6,7].

Within this framework, modeling the lower limb as a damped pendulum allows the estimation of equivalent mechanical parameters, such as stiffness and viscosity, which describe the dynamic response of the passive limb system. These parameters do not represent direct measurements of intrinsic tissue material properties; rather, they provide model-derived estimates reflecting the combined mechanical contribution of joint structures, soft tissues, and segmental inertia during passive oscillations [7,8,9]. Although pendulum-based approaches have been applied to characterize passive joint alterations in neurological and rheumatological conditions, their application to metabolic diseases such as T2DM remains limited.

Previous studies in individuals with diabetes have mainly focused on muscle strength, mobility limitations, and functional performance [1,10,11,12], whereas quantitative assessment of passive joint mechanics has received comparatively little attention. Consequently, it remains unclear whether the multiple musculoskeletal alterations associated with T2DM converge into a measurable change in the integrated passive mechanical behavior of the knee–lower limb system. Investigating this question may provide complementary biomechanical information on diabetes-related musculoskeletal involvement that is not captured by conventional functional assessments.

Accordingly, the primary aim of the present study was to investigate whether individuals with T2DM exhibit differences in the principal kinematic characteristics of the passive knee response compared with healthy controls using an instrumented pendulum test. The primary kinematic outcomes were oscillatory amplitude, angular velocity, relaxation indices, and oscillation duration, assessed through the principal flexion- and extension-related parameters of the pendular response. We hypothesized that T2DM would be associated with reduced pendular excursions and altered temporal characteristics of the passive knee motion.

Model-derived stiffness and viscosity were examined as secondary outcomes, while their variation across successive flexion–extension half-cycles was considered exploratory because the available evidence was insufficient to formulate specific a priori hypotheses regarding differences between groups across individual half-cycles.

Overall, this integrated approach was designed to determine whether passive knee behavior differs between individuals with T2DM and control subjects and whether any between-group differences are reflected primarily in observable kinematic features or are also evident in model-derived mechanical parameters.

## 2. Methods

### 2.1. Participants

A total of 20 participants were included in the final analysis, comprising 10 individuals with type 2 diabetes mellitus (T2DM) and 10 healthy control subjects. Demographic and anthropometric characteristics of the two groups are reported in Table 1A. No statistically significant between-group differences were observed for age, body height, or body mass. Individuals with T2DM were recruited from outpatient clinics and had a confirmed diagnosis of type 2 diabetes mellitus for more than 5 years. Clinical and functional characteristics of the participants with T2DM are reported in Table 1B. All participants were able to walk independently without assistive devices. As part of the functional assessment, all participants performed a 10 m walking test and the Timed Up and Go (TUG) test, with three trials performed for each test. The mean values of the three trials were used for the functional measures reported in Table 1B.

Initially, 15 patients were enrolled; however, five participants were excluded because adequate muscular relaxation could not be achieved, compromising the execution of the pendulum test protocol. Therefore, only participants fulfilling the predefined relaxation criteria required for reliable estimation of model-derived mechanical parameters were included in the final analysis. Healthy controls were recruited from the local community and had no history of diabetes or other major medical conditions affecting lower limb function.

Exclusion criteria for both groups included previous lower limb surgery, neurological disorders, acute musculoskeletal injuries, or any condition potentially affecting normal knee joint mechanics.

All procedures were conducted in accordance with the Declaration of Helsinki. Written informed consent was obtained from all participants prior to data collection. The study protocol was approved by the local institutional ethics committee (Comitato Etico Locale Catania 2, No. 133/CEL, 18 June 2025).

### 2.2. Pendulum Test Protocol and Recording System

**Figure 1:**
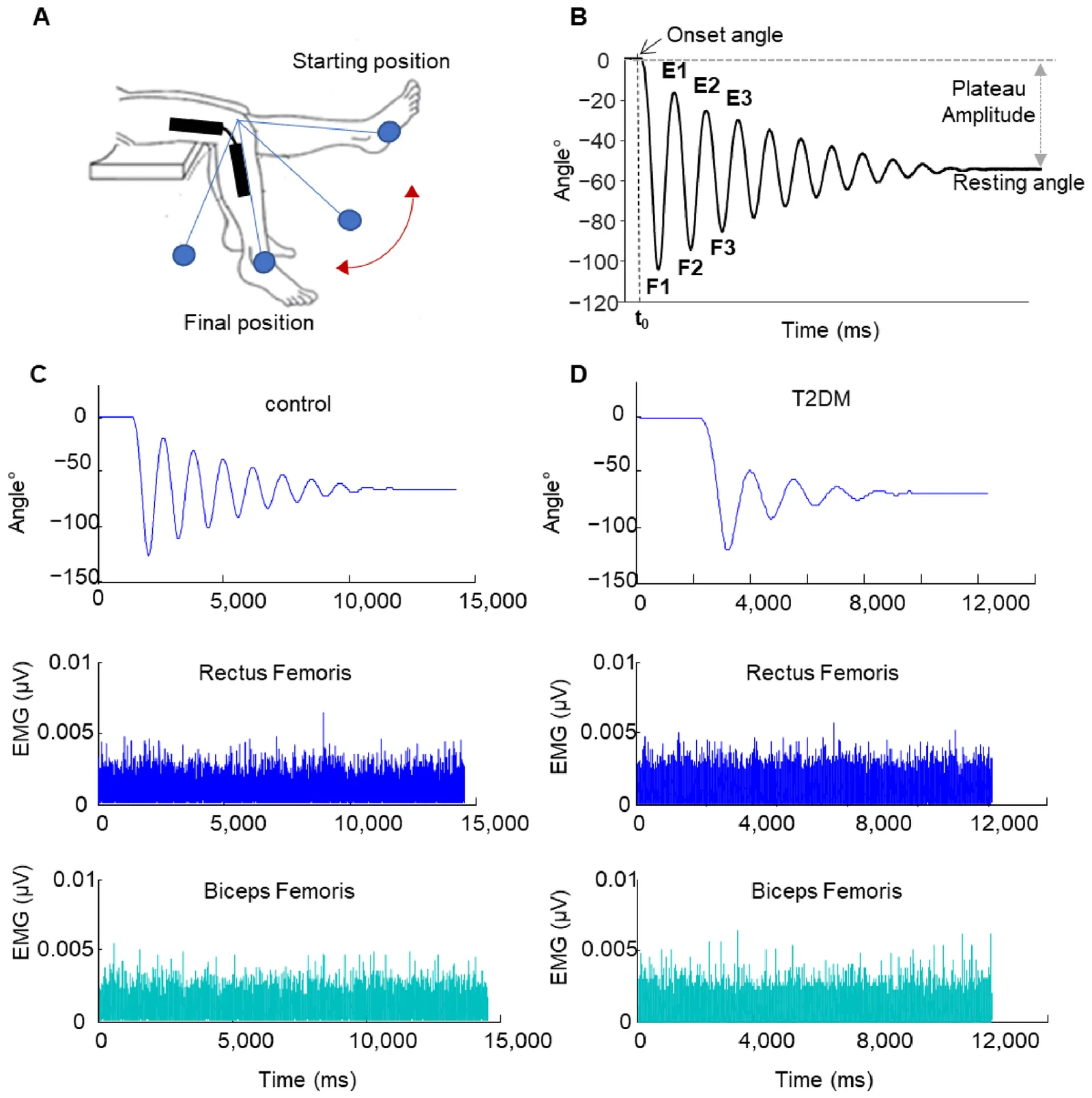
Experimental setup and representative knee joint kinematics and EMG recording during the pendulum test. (**A**) Representation of the experimental configuration and limb positions. The lower limb is shown at the initial position prior to release and at the final resting configuration. (**B**) Representative time course of knee angular displacement during a single pendulum test trial, illustrating the damped oscillatory behavior and the progressive reduction in peak excursion across successive flexion-extension cycles (F1–F3 and E1–E3) until reaching the resting angle. (**C**) Representative knee angular displacement trace from a healthy control subject. (**D**) Representative knee angular displacement trace from a patient with type 2 diabetes mellitus (T2DM).

Passive knee mechanics were assessed using an instrumented pendulum test performed in a standardized semi-seated position (Figure 1A). The trunk was inclined approximately 40° from the horizontal plane to ensure participant comfort and postural stability throughout the procedure.

Participants were instructed to remain as relaxed as possible throughout the procedure and to avoid any voluntary activation or active contribution to the movement of the lower limb muscles. Muscle relaxation was assessed before each trial through visual and palpatory examination and continuously monitored using surface electromyography (EMG) (Bts Bioengineering, 20024 Garbagnate Milanese, Italy).

EMG activity was recorded from the rectus femoris and biceps femoris muscles using bipolar surface electrodes positioned according to surface electromyography for the non-invasive assessment of muscles (SENIAM) recommendations [13]. Electrode placement was verified through inspection of baseline signals. The EMG recordings were used to monitor the absence of detectable voluntary or movement-related muscle activation in the monitored muscles during the oscillatory trials. The rectus femoris and biceps femoris were selected as representative knee extensor and flexor muscles and as indicators of possible antagonist co-contraction. No quantitative EMG threshold was used to define adequate relaxation. EMG recordings were visually inspected for evident voluntary or movement-related muscle activation in conjunction with direct observation of the limb and the resulting oscillatory response. Knee angular motion was recorded using a wearable, biaxial electrogoniometer (SG150, Biometrics Ltd., Newport, UK), positioned across the knee with its two arms aligned with the longitudinal axes of the thigh and lower leg (Figure 1A). The device had a measurement accuracy of ±2° and a repeatability of ±1°. The sensor output was used to reconstruct flexion–extension kinematics throughout each oscillation trial. Kinematic signals were low-pass filtered using a second-order Butterworth filter with a cutoff frequency of 6 Hz, applied in the forward and reverse directions to achieve zero-phase filtering and avoid phase distortion. The filtering was performed before down-sampling and served as the anti-aliasing step. The filtered kinematic signals were subsequently down-sampled from 1000 to 200 Hz for analysis. The resulting Nyquist frequency of 100 Hz was well above the 6 Hz cutoff frequency, ensuring preservation of the retained frequency content without aliasing. Kinematic and EMG signals were synchronously acquired using a portable integrated recording system (FREE-EMG, BTS Bioengineering, Garbagnate Milanese, Italy) at a sampling frequency of 1000 Hz.

The right lower limb was tested in all participants. Before each trial, the examiner manually raised the lower leg and positioned the knee at the maximum passive extension achievable by the participant. The same examiner performed this procedure for all participants to minimize inter-examiner variability. The limb was then released to initiate the pendulum oscillation. No fixed absolute knee angle, predefined angular tolerance, or mechanical release device was used. Each trial consisted of damped flexion–extension oscillations of the knee joint (Figure 1B). A trial was considered valid when, following release, the limb underwent a reproducible passive oscillation without evident active intervention. When the limb remained extended or was actively lowered by the participant after release, the trial was repeated. For participants in whom this occurred repeatedly despite 6–7 attempts, a reproducible passive oscillation could not be obtained, and the participant was excluded from the analysis. Ten valid trials were collected for each participant included in the final analysis.

### 2.3. Kinematic and Model-Derived Mechanical Parameters

Kinematic descriptors of the pendular motion were extracted from each oscillatory trial, including the initial angular position (onset angle), final resting position (resting angle), and the first three peak flexion (F1, F2, F3) and extension (E1, E2, E3) angles (Figure 1B). The primary kinematic outcomes were oscillatory amplitude, angular velocity, relaxation indices, and oscillation duration.

Cycle-specific amplitude parameters were calculated from the identified angular peaks, including first flexion amplitude (F1Amp) = F1 − onset angle, and first extension amplitude (E1Amp) = E1 − F1. Angular velocity parameters were calculated for the corresponding flexion and extension components. Additional kinematic descriptors were calculated to characterize relaxation and the overall organization of the pendular response. Plateau amplitude (PA) was calculated as resting angle–onset angle. Additional normalized descriptors of pendular relaxation were calculated, including the relaxation index (RI): = F1Amp/PA, and extension relaxation index (ERI): = E1Amp/PA.

Temporal characteristics of the movement, including oscillation duration and damping time, were also extracted. To minimize the influence of inter-subject anthropometric variability, kinematic and model-derived mechanical parameters were normalized to the gravitational torque of the leg–foot segment (*mgl_c_*). Anthropometric parameters were estimated according to Winter [14]. Specifically, the segment mass (*m*) was estimated as 6.1% of body mass, while the center of mass distance from the knee joint center (l_c_) was calculated as 60.6% of the segment length. The segment length (*l*) was defined as the distance between the femoral condyle and the medial malleolus. The gravitational torque was then calculated from the estimated *m*, *g*, and *l_c_*.

### 2.4. Estimation of Stiffness and Viscosity Parameters

Anthropometric and kinematic data, together with established computational tables [14], were used to determine the centers of joint rotation and estimate limb mass and acceleration, which were then incorporated into the biomechanical model to derive the viscosity (B) and stiffness (K) coefficients for the first three knee flexion–extension cycles. These model-derived parameters were examined as secondary outcomes to provide complementary information on the mechanical behavior of the passive limb during pendular motion. The coefficients were estimated using a physical pendulum model in which both B and K contributed to the resulting motion, as described by the following equation:

(1) $$J\ddot{\theta }+B\dot{\theta }+K\theta +\mathrm{mg}l_{c}\sin\theta =0$$

where *θ* represented the leg oscillation angle relative to the knee; *J* was the moment of inertia of the leg-foot complex about the axis of rotation around the knee; B was the viscosity coefficient; K was the stiffness coefficient. In Equation (1), the only unknown terms were B and K, whereas all the other parameters were obtained either from the data collected during the experimental procedure or from anthropometric tables [14]. In addition, the same anthropometric tables provided the radius of rotation relative to the center of mass (*ρ*_0_), which, together with *m* and *l_c_*, was required to calculate *J* according to the following equation:

(2) $$J=m\cdot (l_{c}^{2}+\rho_{0\;}^{2})$$

Equation (1) allowed B and K values to be determined for each point along the leg oscillatory trajectory. In this study, B and K were calculated at the first three flexion peaks (half-cycles F1, F2, and F3) and extension peaks (half-cycles E1, E2, and E3). Their calculation was based on their relationship with the damping coefficient (*ζ*) and the natural frequency (*ω_n_*) of the leg oscillations. The coefficient *ζ* depended on the changes in peak amplitude occurring throughout the oscillations, expressed as the ratio between the angular values of two consecutive peaks (D = *θ*F_1_/*θ*F_2_ in the case of flexion peaks), whereas *ω_n_* was derived from the frequency of the peaks. ζ and *ω_n_* were calculated according to the following equations:

(3) $$\zeta =\sqrt{\frac{{(\ln D)}^{2}}{{4\pi^{2}+(\ln D)}^{2}}}$$

(4) $$\omega_{n\;}=\frac{2\pi }{T}$$

where T was the time interval between two consecutive peaks. By entering the experimental data into Equations (3) and (4), *ζ* and *ω_n_* could be calculated.

The relationships among *ζ*, *ω_n_*, B, and K were expressed by the following equations:

(5) $$\zeta =\frac{B}{2J\omega_{n}}$$

(6) $$\omega_{n}^{2}=\frac{K+T_{g}}{J}$$

The values of K and B were then derived from Equations (5) and (6) using the following relationships:

*B* = 2*Jζω_n_* (7)

(8) $$K=J\omega_{n}^{2}-T_{g}$$

For each half-cycle, B and K were determined by modifying Equations (3) and (4), specifically by replacing 4π^2^ with π^2^ in Equation (3) and halving both the numerator and denominator in Equation (4).

### 2.5. Statistical Analyses

Differences in kinematic parameters between T2DM and control subjects were assessed using the Wilcoxon rank-sum test, based on the non-parametric distribution of the data. Analyses were performed using participant-level values, with 10 participants included in each group. To account for multiple comparisons across kinematic parameters, *p*-values were adjusted using the Benjamini–Hochberg false discovery rate (FDR) procedure. Effect size was expressed as the probability of superiority (A), representing the probability that a randomly selected observation from one group exceeds a randomly selected observation from the other group. In addition, a simulation-based post hoc power analysis was performed using the observed group distributions and 1000 simulations. To assess trial-to-trial variability of the principal kinematic measures, the coefficient of variation (CV) was calculated for F1 Amp, E1 Amp, and plateau amplitude (PA) for each participant across the available repeated trials. The CV was calculated as the standard deviation divided by the mean of the repeated-trial values and expressed as a percentage. The resulting participant-level CV values were compared between T2DM and control subjects using the Wilcoxon rank-sum test. *p*-values from the three CV comparisons were adjusted using the Benjamini–Hochberg FDR procedure, and probability of superiority (A) was used as the effect size measure.

To determine whether the diabetic condition altered knee stiffness and viscosity compared with the control condition, and whether these potential changes depended on the Half-Cycle, we carried out a nonparametric factorial analysis using the Aligned Rank Transform (ART) procedure implemented in R by the ARTool package. The ART approach enables the analysis of factorial designs and interaction effects when the normality assumption of a conventional repeated-measures ANOVA may not be satisfied, such as in the present study due to the small sample size [15]. The ART ANOVA model included Group (Control | T2DM) as a between-subject factor and Half-Cycle (F1 | E1 | F2 | E2 | F3 | E3) as a within-subject repeated-measures factor. Fixed effects of Group, Half-Cycle, and their interaction (Group × Half-Cycle) were tested. A Subject ID variable was included as random effect to account for the within-subject dependence of observations. Statistical significance of the fixed effects was set at *p* < 0.05.

Statistical power of the ART ANOVA was assessed using a Monte Carlo simulation approach, by which simulated datasets, based on the distribution parameters of the real data, were generated according to the study design, namely two groups (10 controls and 10 T2DM) and six repeated measurements per subject. For each simulated dataset, the same ART model used in the primary analysis was fitted, and the significance of the Group effect, Half-Cycle effect, and Group × Half-Cycle interaction was evaluated at α = 0.05. Statistical power was estimated as the fraction of simulated datasets yielding, for each fixed effect, a significant result across 1000 simulation iterations. Finally, for the significant fixed-effect factors, we performed pairwise contrasts between levels of the variables using the art.con function of the ARTool package, specifically designed to perform pairwise contrasts within the ART ANOVA framework [16]. Statistical significance of the pairwise contrasts was set at *p* < 0.05, Tukey adjusted.

## 3. Results

### 3.1. Participants

Twenty participants were included in the final analysis, comprising 10 individuals with type 2 diabetes mellitus (T2DM) and 10 healthy controls. Demographic and anthropometric characteristics of the two groups are summarized in Table 1A. No significant between-group differences were observed for age, height, or body mass.

### 3.2. Pendulum-Derived Kinematic and Mechanical Parameters

Kinematic and EMG traces recorded during single trials in one control subject and in one person with T2DM are illustrated in Figure 1C,D. The kinematic traces (the top panel in columns C and D) provide a representative visualization of the knee motion during the pendulum test. The corresponding EMG traces (the middle and bottom panels of the same columns) show the activity of the rectus femoris and biceps femoris muscles monitored throughout the procedure. We first assessed the trial-to-trial variability of the principal kinematic measures by computing the coefficient of variation (CV) for F1 Amp, E1 Amp, and plateau amplitude (PA) for each participant across the available pendulum test trials. The resulting CV values were then compared between participants with T2DM and controls (Table 2). The CV values were subsequently compared between T2DM and control subjects using the Wilcoxon rank-sum test, with Benjamini–Hochberg correction applied to the three comparisons. No significant between-group differences were observed for F1 Amp CV (W = 50, *p* = 1.000, p_FDR = 1.000, probability of superiority A = 0.50), E1 Amp CV (W = 39, *p* = 0.427, p_FDR = 1.000, A = 0.61), or PA CV (W = 48, *p* = 0.910, p_FDR = 1.000, A = 0.52). Thus, the trial-to-trial variability of these principal kinematic measures did not differ significantly between T2DM and control subjects.

**Figure 2:**
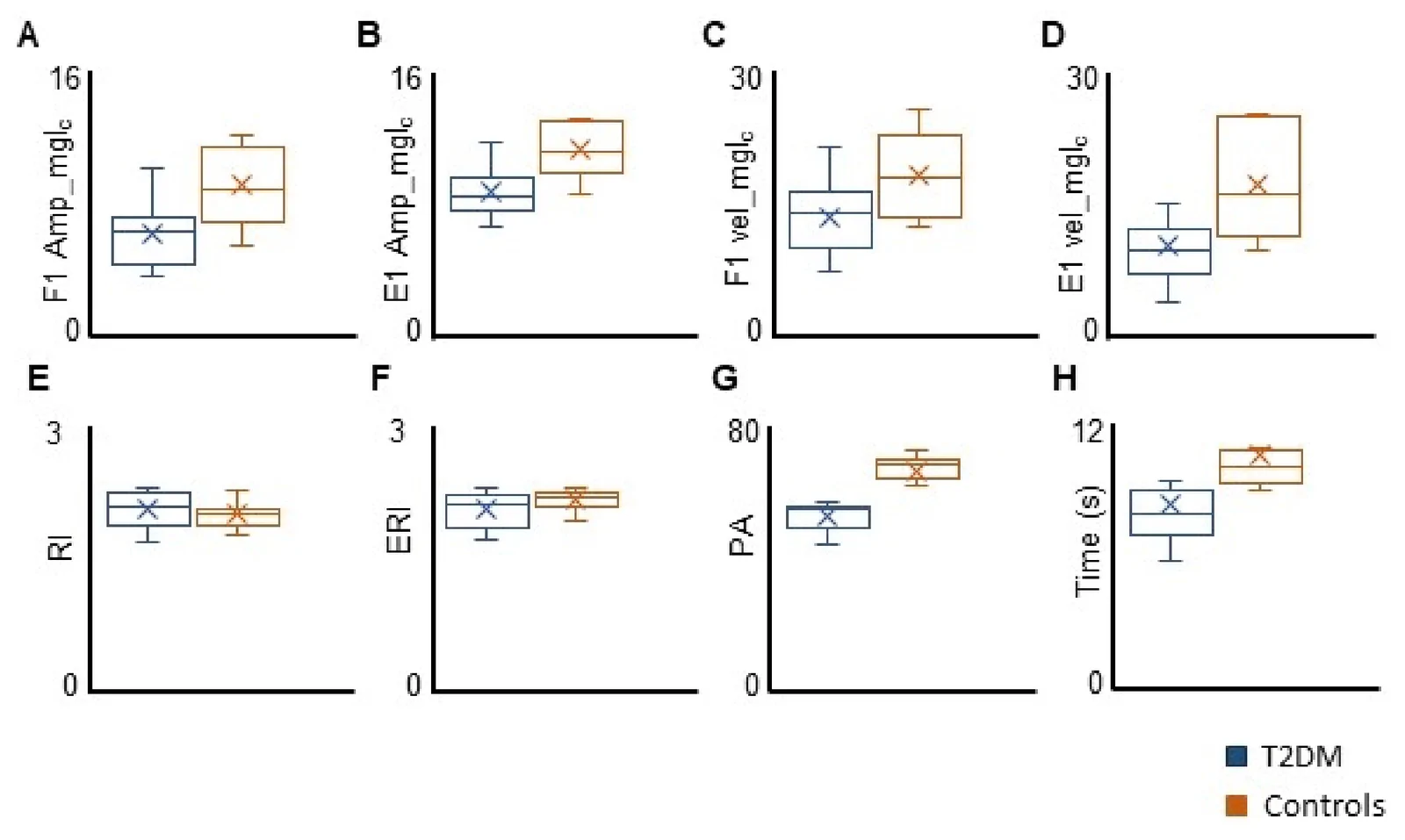
Kinematic responses during the instrumented pendulum test. Knee kinematic characteristics represented as box-and-whisker plots, obtained from the instrumented pendulum test in patients with type 2 diabetes mellitus, and healthy controls (orange). Panels (**A**,**B**) show knee angular amplitudes in flexion and extension, panels (**C**,**D**) show their peak angular velocities, panels (**E**,**F**) show relaxation indices, panel (**G**) shows the plateau amplitude (PA), and panel (**H**) shows the total duration of the oscillations. Box-and-whisker plots report the median of the distribution of the mean values among trials observed in each participant (horizontal line inside the box), the interquartile range as the vertical length of the box, the highest and lowest values as the whiskers, as well as the mean value across subjects with the (X) symbol.

The between-group distributions of the selected passive knee kinematic parameters are shown in Figure 2, providing a visual representation of the variability between T2DM and control subjects. Quantitative between-group comparisons are reported in Table 3. After Benjamini–Hochberg FDR correction, significant differences were observed for F1Amp_mgl_c_, E1Amp_mgl_c_, E1vel_mgl_c_, PA, and DUR, whereas F1vel_mgl_c_, RI, and ERI did not reach statistical significance.

Among the significant parameters, PA showed the strongest between-group separation, with the highest probability of superiority (A = 0.94) and post hoc power (99.2%). E1Amp_mglc, F1Amp_mglc, E1vel_mglc, and DUR also showed a relatively large between-group separation, with probability of superiority values ≥0.82. RI and ERI showed limited between-group separation and low post hoc power, whereas F1vel_mglc showed an intermediate probability of superiority but did not reach statistical significance after FDR correction.

### 3.3. Knee Stiffness and Viscosity in T2DM and Control Subjects

**Figure 3:**
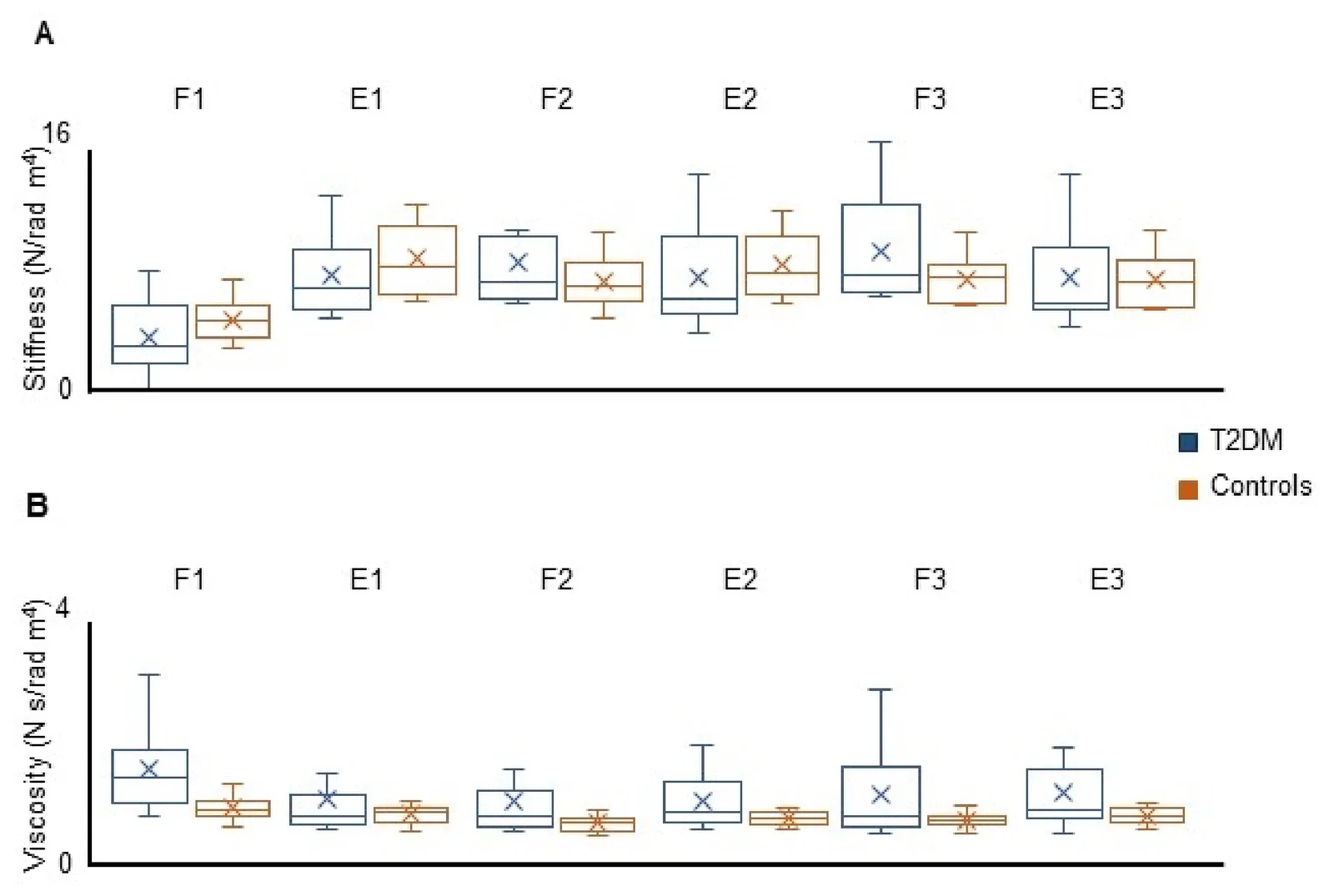
Knee stiffness and viscosity estimated during the Half-Cycle phases in control and T2DM subjects. (**A**) Distribution of knee stiffness values across the different Half-Cycle phases in healthy controls (orange) and patients with T2DM. (**B**) Corresponding distributions of knee viscosity values. Box-and-whisker plots represent participant-level mean values across trials; the horizontal line within each box indicates the median, the box represents the interquartile range, and the whiskers indicate the minimum and maximum values. The × symbol denotes the mean value across participants.

The distributions of knee stiffness and viscosity estimates across the different Half-Cycle phases in T2DM and control subjects are illustrated by the box plots in Figure 3.

To assess potential between-group differences and changes across Half-Cycle phases, ART ANOVA models were fitted including Group, Half-Cycle, and their interaction as fixed effects. The results of the ART ANOVA models are summarized in Table 4 and Table 5 for the stiffness and viscosity estimates, respectively.

For stiffness, Half-Cycle was the strongest factor explaining the observed variation, with a marked increase in the stiffness values between F1 and E1 (pairwise contrast: t-ratio_(90)_ = 13.09, *p* < 0.0001; see also Figure 3A). No significant overall Group effect was observed. However, a significant Group × Half-Cycle interaction indicated that the phase-dependent pattern of model-derived stiffness differed between T2DM and control subjects across the pendular response. Post hoc comparisons at individual half-cycles did not reveal significant between-group differences after correction for multiple comparisons (all pairwise contrasts, *p* > 0.05). Thus, although the overall interaction was statistically significant, it could not be attributed to a significant T2DM–control difference at any specific half-cycle.

Regarding the knee viscosity, a significant Half-Cycle effect was also observed, together with a significant Group × Half-Cycle interaction, whereas the overall Group effect was not significant. Post hoc comparisons at individual half-cycles did not reveal significant between-group differences after correction for multiple comparisons. Thus, as for stiffness, the significant interaction indicates a difference in the overall phase-dependent pattern of model-derived viscosity across the pendular response, without identifying a specific half-cycle at which T2DM and control subjects differed significantly.

## 4. Discussion

The present study provides evidence of differences in passive knee behavior between individuals with T2DM and control subjects during pendular motion. By combining conventional kinematic descriptors with model-derived mechanical parameters, the instrumented pendulum test captured complementary aspects of the passive knee response, including oscillatory excursions, angular velocity, oscillation duration, and the temporal organization of passive mechanical behavior across the oscillatory cycle. These findings indicate that T2DM is associated primarily with reduced passive oscillatory excursions, whereas model-derived stiffness and viscosity did not show overall between-group differences despite significant variation across successive half-cycles and significant Group × Half-Cycle interactions. Importantly, these interactions were not explained by significant between-group differences at individual half-cycles after correction for multiple comparisons. Together, these findings support the potential value of integrating kinematic analysis with biomechanical modeling to characterize system-level differences in passive musculoskeletal behavior associated with T2DM.

The reduced normalized F1Amp and E1Amp observed in individuals with T2DM indicate differences in passive knee oscillatory kinematics during pendular motion. The pendulum test evaluates the response of a limb segment released under gravity and provides an integrated assessment of passive kinematic behavior by reflecting the combined influence of limb inertia, joint properties, and viscoelastic resistance of periarticular musculoskeletal tissues [6,7,9,17]. Within this biomechanical framework, the reduced oscillatory excursions observed in the T2DM group suggest a less compliant kinematic response of the musculoskeletal system, consistent with increased resistance to passive motion. Because the test was performed with continuous electromyographic monitoring of the rectus femoris and biceps femoris to detect voluntary muscle activation, the observed alterations are interpreted as primarily reflecting changes in the system-level passive mechanical behavior of the knee–lower limb system, although low-level activation of unmonitored muscles cannot be completely excluded [9,17]. Collectively, reduced oscillation amplitude, lower extension angular velocity, and shorter oscillation duration indicate an altered passive knee response in individuals with T2DM.

Previous studies have shown that altered passive mobility in diabetes involves multiple anatomical regions and is closely associated with metabolic remodeling of connective tissues. Among the mechanisms proposed to explain these alterations, the accumulation of AGEs has received considerable attention. AGE-mediated non-enzymatic collagen cross-linking can alter collagen fibril organization, intermolecular sliding, energy dissipation, and mechanical behavior, thereby contributing to impaired tissue compliance and joint mobility [4,18,19,20].

Although the present study did not directly assess collagen composition or AGE accumulation, these well-established structural alterations provide a biologically plausible framework for interpreting the reduced passive oscillatory behavior observed in individuals with T2DM. Clinically, diabetes-associated connective tissue remodeling manifests as reduced range of motion and increased stiffness of periarticular structures, characteristic of limited joint mobility syndrome [21,22]. Thus, the reduced amplitude and extension velocity observed during passive pendular motion may be compatible with the broader pattern of reduced tissue compliance and joint mobility reported in diabetes.

Additional kinematic descriptors provided further information on the passive pendular response. Plateau amplitude (PA) was significantly reduced in individuals with T2DM compared with control subjects, indicating a marked reduction in the overall magnitude of the pendular excursion. This finding is consistent with the reduced F1Amp and E1Amp observed in the T2DM group and further supports the presence of a reduced passive oscillatory response. In contrast, although the extension relaxation index (ERI) was numerically lower in individuals with T2DM than in controls, this difference was not statistically significant. Similarly, no significant between-group difference was observed for the relaxation index. These findings suggest that the between-group differences in passive pendular behavior are primarily related to the magnitude of the oscillatory excursion rather than to a consistent alteration in the relative relaxation indices. Importantly, the absence of significant between-group differences in the coefficient of variation in F1 Amp, E1 Amp, and PA suggests that the observed differences in these principal kinematic measures were not accompanied by a detectable difference in trial-to-trial variability. This finding supports the interpretation that the between-group differences primarily reflect differences in the magnitude of passive knee excursion rather than greater variability across repeated trials in participants with T2DM. The shorter oscillation duration observed in participants with T2DM further supports the presence of altered temporal characteristics of the passive pendular response. The progressive decay of oscillatory motion reflects the interplay between inertial properties, restoring forces, and energy dissipation within the musculoskeletal system. In biomechanical systems, these components are commonly represented through second-order models, where stiffness and damping parameters describe the dynamic response of the limb during pendular motion [7,9,17]. However, model-derived stiffness and viscosity estimates should be interpreted as descriptors of the overall passive mechanical behavior of the system rather than direct measurements of individual tissue material properties.

Model-derived stiffness and viscosity were examined as secondary outcomes to provide complementary information on passive limb mechanics.

No overall between-group difference was observed for model-derived stiffness, indicating that T2DM was not associated with a uniform increase or decrease in stiffness across the pendular response. Stiffness varied significantly across successive flexion–extension half-cycles, and a significant Group × Half-Cycle interaction was also observed (Table 4). However, the interaction was not explained by significant between-group differences at individual half-cycles after correction for multiple comparisons. Therefore, these findings cannot be interpreted as evidence of a specific phase-dependent increase or decrease in stiffness in T2DM. Given the secondary and exploratory nature of this analysis, this finding should be considered hypothesis-generating and requires confirmation in larger cohorts. The variation in stiffness across half-cycles is biomechanically plausible because passive joint resistance may vary according to joint position, muscle length, and the mechanical contribution of different passive structures [23]. During pendular motion, the relative contribution of these components may therefore change as the limb moves through successive flexion and extension excursions. Accordingly, evaluating stiffness across successive oscillation phases may provide complementary information on passive mechanical behavior that cannot be captured by a single global estimate. Model-derived viscosity was also examined as a secondary outcome to provide complementary information on the damping behavior of the passive limb. No significant overall between-group difference in viscosity was observed, indicating that T2DM was not associated with a uniform alteration in viscosity across the pendular response. Viscosity varied significantly across successive flexion–extension half-cycles, and a significant Group × Half-Cycle interaction was also observed (Table 5). However, as for stiffness, the interaction was not explained by significant between-group differences at individual half-cycles after correction for multiple comparisons. Accordingly, the significant interaction indicates that the phase-dependent pattern of model-derived viscosity differed statistically between groups across the pendular response, but does not provide evidence of a T2DM-control difference at any specific half-cycle. Given the exploratory nature of the half-cycle analysis and the small sample size resulting in a relatively low statistical power of the interaction effect, this finding should be considered preliminary and requires confirmation in larger cohorts. These observations are consistent with current evidence indicating that T2DM affects multiple components of the musculoskeletal system, including skeletal muscle, connective tissues, tendons, and joint function, through interacting metabolic, inflammatory, and extracellular matrix alterations [21,22,24].

The present findings may have clinical relevance, as reduced passive joint excursions and altered kinematic responses could contribute to movement limitations and functional decline commonly observed in older adults with T2DM. Diabetes-related musculoskeletal impairment is increasingly recognized as a multifactorial condition involving skeletal muscle dysfunction, impaired muscle quality, connective tissue remodeling, and reduced joint mobility [11,22,25,26]. In this context, recent molecular evidence suggests that T2DM-associated sarcopenia involves interacting mechanisms related to impaired energy metabolism, mitochondrial dysfunction, inflammatory signaling, and altered muscle homeostasis [10,27].

Although the current investigation was not designed to assess these molecular mechanisms directly, the observed differences in passive knee behavior are compatible with a multifactorial contribution of metabolic, structural, and functional factors to musculoskeletal dysfunction in T2DM.

Several limitations should be acknowledged. First, the sample size was relatively small, although the study protocol required careful participant selection and repeated attempts to obtain a reproducible passive pendulum response. Second, five of the 15 initially recruited participants with T2DM (33.3%) were excluded because a reproducible passive pendulum oscillation could not be obtained despite several attempts per participant. This exclusion may have introduced selection bias, as the final sample may preferentially represent individuals with T2DM who are able to adequately comply with the passive pendulum procedure. Therefore, the findings should be generalized primarily to individuals with T2DM who are able to perform a reproducible passive pendulum test. Third, although Table 1B now provides individual clinical and functional information, including glycemic control and metabolic status, as well as functional measures such as comfortable walking speed and TUG test duration, some potentially relevant musculoskeletal conditions, particularly osteoarthritis, were not systematically documented for all participants. Although the TUG test provides an overall measure of functional mobility, specific musculoskeletal conditions affecting joint mobility were not systematically assessed. These factors may independently influence passive knee kinematics and therefore represent potential sources of residual confounding. Although individual metabolic measures are now reported in Table 1B, the small sample size did not allow us to adequately investigate whether the observed kinematic differences were related to the degree of glycemic control or other metabolic characteristics. Therefore, the observed between-group differences should be interpreted as associations with T2DM status rather than as evidence of a specific effect of glycemic control or other individual metabolic factors. Fourth, stiffness and viscosity were estimated using a biomechanical model incorporating anthropometric assumptions and should be interpreted as descriptors of system-level mechanical behavior rather than direct measurements of the mechanical properties of individual tissues. Finally, only the right lower limb was evaluated.

Future studies incorporating bilateral assessments, larger and clinically stratified cohorts, and integration with metabolic, imaging, and tissue-specific measurements will be important to further clarify the underlying mechanisms and to determine whether the observed alterations extend to the broader T2DM population, including individuals who may have difficulty performing a reproducible passive pendulum test. Overall, the present study indicates that individuals with T2DM exhibit differences passive knee kinematics detectable through instrumented pendulum testing, primarily characterized by reduced passive oscillatory excursions and altered temporal characteristics. Model-derived stiffness and viscosity did not show significant overall between-group differences but exhibited significant phase-dependent Group × Half-Cycle interactions that could not be localized to specific half-cycles. These secondary exploratory findings require confirmation in larger cohorts. By integrating kinematic descriptors with model-derived mechanical parameters, instrumented pendulum testing may provide complementary, non-invasive information on system-level passive knee behavior associated with T2DM.

## 5. Conclusions

The present study indicates that type 2 diabetes mellitus is associated with measurable differences in passive knee behavior during pendular motion, particularly characterized by reduced oscillatory excursions and altered temporal characteristics of the movement. Model-derived stiffness and viscosity provided complementary information on the system-level mechanical response of the passive limb. Both parameters varied across successive flexion–extension half-cycles and showed significant Group × Half-Cycle interactions; however, these interactions were not explained by significant between-group differences at individual half-cycles after correction for multiple comparisons. Therefore, the present findings do not provide evidence of a specific phase at which T2DM-related differences in model-derived stiffness or viscosity can be identified. Further integration of instrumented pendulum testing with metabolic, structural, and tissue-specific investigations may enhance our understanding of the biological mechanisms underlying diabetes-related alterations in passive musculoskeletal function.

## Figures and Tables

**Table 1:** (**A**). Anthropometric characteristics of the study population. (**B**). Individual clinical and functional characteristics of participants with T2DM.

**A**							
**Variable**		**Controls** **(n = 10)**		**T2DM** **(n = 10)**		***p* Value**	
Age (years)		71.8 ± 7.6		76.2 ± 6.4		0.13	
Height (cm)		167.0 ± 6.9		163.0 ± 12.2		0.58	
Body mass (kg)		71.9 ± 10.4		71.4 ± 11.3		0.72	
Gender		♂ = 6		♂ = 6			
**B**							
**Subj**	**Diabetes Duration (years)**	**PRE** **(mg/dL)**	**POST** **(mg/dL)**	**HbA1c (%)**	**Homa-IR**	**Walking** **Speed** **(m/s)**	**TUG** **Duration** **(s)**
01	7	128 ± 15.2	164 ± 21.4	7.1	3.91	0.96 ± 0.08	11.5 ± 0.3
02	9	119 ± 18.6	153 ± 24.1	6.8	3.11	0.70 ± 0.05	14.8 ± 0.5
03	8	142 ± 20.3	181 ±27.6	7.4	6.16	0.92 ± 0.09	13.0 ± 0.2
04	35	126 ± 17.8	158 ±22.9	6.9	2.87	1.20 ± 0.13	10.8 ± 1.3
05	26	138 ± 22.1	176 ± 29.3	6.6	4.05	1.10 ± 0.09	13.3 ± 0.3
06	17	112 ± 14.7	146 ± 19.8	7.7	2.31	0.78 ± 0.12	17.2 ± 0.5
07	8	131 ± 19.5	169 ± 25.7	7.2	2.81	1.11 ± 0.08	11.6 ± 0.4
08	23	145 ± 23.4	190 ± 31.2	7	3.76	1.00 ± 0.07	11.9 ± 0.3
09	18	124 ± 16.1	159 ± 20.6	6.7	3.5	1.52 ± 0.03	10.1 ± 0.5
10	28	136 ± 18.9	174 ± 26.3	7.3	4.65	1.04 ± 0.15	13.0 ± 0.4

Footnote: values are mean ± SD. Note: PRE = Pre-prandial blood glucose; POST = Post-prandial blood glucose. Pre- and postprandial blood glucose values represent the mean ± standard deviation values recorded over the year preceding the experimental evaluation. HbA1c = glycated hemoglobin. Walking speed and Timed Up and Go (TUG) test values are reported as the mean ± standard deviation of three trials performed at comfortable walking speed and during the TUG test, respectively.

**Table 2:** Trial-to-trial variability of the principal kinematic parameters in T2DM and control subjects.

	F1Amp		E1Amp		PA	
	T2DM	Controls	T2DM	Controls	T2DM	Controls
median	5.92	5.05	12.23	8.02	4.25	4.17
quartile 1	2.88	4.59	6.08	6.79	3.60	3.52
quartile 3	8.64	5.77	18.31	9.27	4.91	4.92

Note: Values represent the median, first quartile (Q1), and third quartile (Q3) of the coefficient of variation (CV) calculated across available pendulum test trials for each participant. CV values are reported for first flexion amplitude (F1Amp), first extension amplitude (E1Amp), and plateau amplitude (PA), separately for participants with type 2 diabetes mellitus (T2DM) and control subjects.

**Table 3:** Between-group comparison of selected passive knee mechanical parameters assessed by the pendulum test.

Variable	W	FDR-Adjusted *p*	A	Post Hoc Power (%)
F1Amp_*mgl_c_*	18	**0.0294**	0.82	77.1
E1Amp_*mgl_c_*	12	**0.0096**	0.88	93.0
F1vel_*mgl_c_*	24	**0.0874**	0.76	55.2
E1vel_*mgl_c_*	18	**0.0294**	0.82	77.8
RI	59	0.6611	0.59	11.9
ERI	36	0.4500	0.64	19.9
PA	6	**0.0032**	0.94	99.2
DUR	12	**0.0096**	0.88	90.5

Note: Data are presented as Wilcoxon rank-sum test statistics (W). *p*-values were adjusted for multiple comparisons using the Benjamini–Hochberg false discovery rate (FDR) procedure. Probability of superiority (A) represents the magnitude of between-group separation, expressed as the probability of superiority of the group with higher values; values of 0.50 indicate no between-group separation, whereas values closer to 1.00 indicate greater separation. Post hoc power was estimated by Monte Carlo simulation (1000 simulations) based on the observed group distributions, with α = 0.05 and a two-sided test. In bold, FDR-adjusted *p*-value < 0.05, considered statistically significant.

**Table 4:** ART ANOVA results for knee stiffness across Half-Cycle phases in Control and T2DM subjects.

**Half-Cycle**				**Controls**	**T2DM**
F1				4.28 ± 0.39	2.97 ± 0.72
E1				8.05 ± 0.71	7.03 ± 0.83
F2				6.54 ± 0.51	7.83 ± 0.99
E2				7.61 ± 0.63	6.88 ± 0.99
F3				6.77 ± 0.48	8.55 ± 1.08
E3				7.22 ± 0.53	7.35 ± 0.95
**FACTOR**	**DoF**	**F**	**Pr (>F)**	**η^2^p**	**Power**
group	1, 18	1.16	0.2949	0.06	6.90%
half-cycle	5, 90	52.47	<0.0001	0.74	99.80%
group × half-cycle	5, 90	8.03	<0.0001	0.31	43.80%

Footnote: DoF = Degrees of Freedom; F = F-statistic; Pr(>F) = Probability > F; (η^2^p) = partial eta squared. Values are reported as mean ± standard error.

**Table 5:** ART ANOVA results for knee viscosity across Half-Cycle phases in Control and T2DM subjects.

**Half-Cycle**				**Controls**	**T2DM**
F1				0.82 ± 0.06	1.42 ± 0.20
E1				0.72 ± 0.05	0.96 ± 0.22
F2				0.57 ± 0.04	0.92 ± 0.19
E2				0.65 ± 0.03	0,91 ± 0.13
F3				0.63 ± 0.04	1.04 ± 0.24
E3				0.62 ± 0.04	1.08 ± 0.21
**FACTOR**	**DoF**	**F**	**Pr (>F)**	**η^2^p**	**Power**
group	1, 18	2.14	0.1604	0.06	30.90%
half-cycle	5, 90	18.15	<0.0001	0.74	55.10%
group × half-cycle	5, 90	4.50	0.0010	0.31	13.30%

Footnote: DoF = Degrees of Freedom; F = F-statistic; Pr(>F) = Probability > F; (η^2^p) = partial eta squared. Values are reported as mean ± standard error.

## Data Availability

The data presented in this study are available on request from the corresponding author. The data are not publicly available due to privacy restrictions.
